# Iron in Child Obesity. Relationships with Inflammation and Metabolic Risk Factors

**DOI:** 10.3390/nu5062222

**Published:** 2013-06-19

**Authors:** Dominique Bouglé, Jacques Brouard

**Affiliations:** Pediatric Department, CHU de Caen, 14033 Caen cedex, France; E-Mail: brouard-jacques@chu-caen.fr

**Keywords:** iron, obesity, children, inflammation, metabolic syndrome, non alcoholic fatty liver disease (NAFLD)

## Abstract

Iron (Fe) sequestration is described in overweight and in its associated metabolic complications, *i.e.*, metabolic syndrome (MetS) and non-alcoholic liver fatty disease (NAFLD); however, the interactions between Fe, obesity and inflammation make it difficult to recognize the specific role of each of them in the risk of obesity-induced metabolic diseases. Even the usual surrogate marker of Fe stores, ferritin, is influenced by inflammation; therefore, in obese subjects inflammation parameters must be measured together with those of Fe metabolism. This cross-sectional study in obese youth (502 patients; 57% girls): 11.4 ± 3.0 years old (x ± SD); BMI z score 5.5 ± 2.3), multivariate regression analysis showed associations between Fe storage assessed by serum ferritin with risk factors for MetS and NAFLD, assessed by transaminase levels, which were independent of overweight and the acute phase protein fibrinogen. Further studies incorporating the measurement of complementary parameters of Fe metabolism could improve the comprehension of mechanisms involved.

## 1. Introduction

Alterations of iron (Fe) metabolism can occur in overweight and obese children and adults [1,2,3,4,5,6,7,8,9,10]. Obesity is a low-grade inflammatory disease which may increase Fe tissue storage at the expense of circulating blood Fe, potentially leading to tissue overload and decreased of Fe available for hematopoiesis. Tissue Fe sequestration is associated with the occurrence of the so-called metabolic syndrome (MetS) which clusters obesity, hypertension, insulin resistance or type 2 diabetes and dyslipoproteinemia; it is also linked to the development of nonalcoholic fatty liver disease (NAFLD), which is the hepatic manifestation of the MetS [11,12,13,14]. Owing to the interactions between obesity, Fe and inflammation, it is difficult to disentangle these parameters given that several Fe parameters, mainly serum Fe and its binding protein transferrin are altered by inflammation. Some have suggested the measurement of other parameters of Fe status and regulation such as soluble transferring receptor (sTfr) and hepcidin, the major regulator of systemic regulation [15,16,17], however, their use in clinical practice is limited [7,10,17,18,19]. However, serum ferritin, a marker of tissue Fe storage, although an inflammatory protein, is currently considered as the best clinical indicator for detecting iron deficiency in the absence of inflammation or in conjunction with the assessment of an inflammatory protein [15,16].

The present report assessed the independent relationships that Fe status, inflammation, and overweight have with the risk factors for MetS and NAFLD in obese youth; serum ferritin measurement was used to assess Fe status as recommended [15,16]. Transaminase levels were used as a marker of potential early NAFLD [12].

## 2. Experimental Section

The study is part of a research protocol on child obesity, which has been approved by the review board of the Hôtel Dieu Hospital (Paris, France); parents and children gave their signed consent to the study.

Subjects attending the specialized outpatient clinic of the University teaching Hospital of Caen for overweight were screened before initiation of behavioral eating and physical activity intervention.

Clinical examination of children was performed by the same clinician. Subjects were apparently healthy. Puberty was assessed by Tanner stages of pubic hair. Weight was assessed with an electronic beam balance; height was measured twice to the nearest 0.5 cm.

Patients included were clinically healthy, without acute infection, and did not take drugs known to induce weight changes; they had given an informed consent to the check-up. Overweight was defined as a BMI over two SDs from the mean of French charts [20].

Blood pressure (BP) was measured by an automatic device.

Blood was drawn after an overnight fast, for measurements of blood glucose, insulin, triglycerides, HDL and LDL cholesterol, transaminases (ALAT, ASAT), fibrinogen, ferritin, hemoglobin and hematocrit. Insulin resistance was assessed by calculating the HOMA-IR (Insulin (mU) × Glycemia (mmol)/22.5). Serum ferritin cut-off levels were set at 12 and 15 mg/L, since both values have been recommended [15,16].

### Statistical Analysis

Results were expressed as mean ± SD.

Simple regression analyses were performed between ferritin and metabolic risk factors, followed by multiple regression analysis including z score, inflammation, sex and age as covariates.

Statistical significance level was set at *p* < 0.05.

## 3. Results

Five hundred and two obese subjects (57% girls) were studied.

Characteristics of these patients are given in Table 1; simple regression (Table 2) displayed a significant correlation between ferritin and fibrinogen, triglycerides, LDL cholesterol, transaminase levels, hemoglobin and hematocrite; between z score and systolic BP fibrinogen, glycemia, HOMA IR, triglycerides, HDL cholesterol, and transaminase levels.

**Table 1:** Characteristics of patients.

	All group	Girls	Boys
*N* (% girls)	502 (57%)		
Age (y)	11.4 ± 3.0 *	11.4 ± 3.2	11.5 ± 2.8
Tanner stage	2.4 ± 1.7	2.8 ± 1.9	1.9 ± 1.4 ^a^
Z score	5.5 ± 2.3	5.0 ± 2.3	5.8 ± 2.6 ^a^
Ferritin (mg/L)	41.7 ± 22.9	39.2 ± 20.7	45.3 ± 26.7 ^a^
Ferritin < 12 mg/L (%)	2.0	2.8	0.8
Ferritin < 15 mg/L (%)	4.8	6.0	2.1 ^a^
Fibrinogen (g/L)	3.6 ± 0.7	3.6 ± 0.8	3.5 ± 0.7
Systolic BP (mm Hg)	116 ± 15	115 ± 17	119 ± 13 ^a^
Diastolic BP (mmHg)	63 ± 12	62 ± 13	63 ± 12
Glycemia (mmol/L) **	4.8 ± 0.4	4.7 ± 0.4	4.9 ± 0.4 ^a^
HOMA IR **	2.3 ± 1.64	2.2 ± 1.4	2.7 ± 2.0 ^a^
Serum triglycerides (mmol/L) **	0.39 ± 0.62	0.86 ± 0.56	0.98 ± 0.70 ^a^
HDL Cholesterol (mmol/L) **	1.44 ± 0.35	1.44 ± 0.36	1.40 ± 0.37
LDL Cholesterol (mmol/L) **	2.69 ± 0.46	2.67 ± 0.74	2.66 ± 0.76
ASAT (IU/L)	24 ± 7	23 ± 8	25 ± 8 ^a^
ALAT (IU/L)	25 ± 7	22 ± 15	27 ± 16 ^a^
Hemoglobin (g/dL)	13.1 ± 1.0	13.0 ± 0.8	13.3 ± 1.1 ^a^
Hematocrite (%)	38.7 ± 3.1	38.6 ± 2.2	39.3 ± 3.3 ^a^

* mean ± SD; ** fasting values; ^a^ different from girls (*p* < 0.05).

Multivariate regression analysis (Table 3) showed that when corrected for sex and age, independent correlations were found between ferritin and triglycerides, HDL cholesterol, transaminase levels and hemoglobin and hematocrite; between z score and systolic BP, blood glucose, HOMA IR, triglycerides, HDL cholesterol, ASAT, and ALAT, and between fibrinogen and HDL cholesterol, ASAT and hematocrite.

**Table 2:** Simple regressions between ferritin and overweight and metabolic risk parameters in obese patients.

	Ferritin		Z score	
	F	*p*	F	*p*
Ferritin			<0.01	0.994
Systolic BP	0.02	0.896	6.87	0.009
Diastolic BP	2.69	0.102	1.76	0.185
Fibrinogen	6.41	0.012	18.65	<0.001
Glycemia *	0.89	0.346	11.90	<0.001
HOMA IR	8.32	0.087	50.81	<0.001
TG *	11.87	<0.001	23.45	<0.001
HDL *	3.61	0.058	31.73	<0.001
LDL	5.08	0.013	1.93	0.165
ASAT	15.47	<0.001	6.64	0.010
ALAT	42.32	<0.001	28.69	<0.001
Hemoglobin	14.37	<0.001	0.02	0.888
Hematocrite	12.04	<0.001	0.29	0.591

* fasting values.

**Table 3:** Multivariate analysis between ferritin and metabolic risk factors corrected for sex, age, z score, and fibrinogen as covariates, in obese patients. (LDL did not enter the model: F = 2.04; *p* = 0.073).

Dependent variables			Covariates				
			Sex	Age	Ferritin	z score	Fibrinogen
	F	*p*	*p*	*p*	*p*	*p*	*P*
Systolic BP	9.36	<0.001	0.319	<0.001	0.208	0,001	0.217
Diastolic BP	3.04	0.011	0.836	0.004	0.149	0.195	0.410
Glycemia	6.14	<0,001	<0.001	0.003	0,485	0.008	0.603
HOMA IR	19.27	<0.001	0.839	<0.001	0.153	<0.001	0.988
Plasma TG	8.35	<0,001	0.962	0.005	<0.001	<0.001	0.440
HDL Cholesterol	10.80	<0.001	0.272	<0.001 *	0.007 *	<0.001 *	0.004
ASAT	13.76	<0.001	0.060	<0.001 *	<0.001	0.001	0.024 *
ALAT	20.27	<0.001	0.057	0.016	<0.001	<0.001	0.076
Hemoglobin	9.02	<0.001	0.109	<0.001	<0.001	0.083	0.058
Hematocrite	10.645	<0.001	0.092	<0.001	0.001	0.488	0.046 *

* negative correlation.

## 4. Discussion

Alterations of iron status have been described in child obesity [1,2,3,4,5,6,7,8,9,10]; inflammation is associated with a shift of Fe from blood, decreasing serum Fe and transferrin levels; so when they are used as surrogate markers of Fe status, an increased prevalence of deficiency is found in overweight subjects [1,6,9]; on the other hand ferritin is usually normal or increased in these patients [3,9,10]; measurement of other parameters of Fe metabolism such as hepcidin did not contribute to the diagnosis [7,10].

Our population of obese youth displayed a prevalence of Fe deficiency of 2.0%–4.8%, (depending on the cutoff level of ferritin) similar to or even lower than large surveys of healthy French youth: in the review by Hercberg *et al.* 13.6% of 2–6 years old French children, and 3.1%–15.4% of adolescent girls had abnormal serum ferritin [21]. Similar values (13.1% low ferritin values) were observed for adolescents with a similar way of life, such as Canadian adolescents [22]. As expected, girls were more prone to be deficient than boys. Other studies reported a similar prevalence of biological Fe deficiency (11.2%) in obese adolescents [6,9]; contrary to the present data, the prevalence of iron deficiency was reported to be increased in overweight patients; however other factors than overweight itself could explain these observations since the prevalence does not seem to be weight dependent: for Nead *et al.* it increased from 2.1% in normal weight to 5.3%, and 5.5% in overweight and obese children, respectively [2]; for others, gender was a main determinant of Fe deficiency in obese adolescents [4].

Defining the precise roles of iron and obesity in the occurrence of obesity-associated metabolic risk factors is a difficult task, owing to the enhancing interactions between oxidative stress and inflammation, which enhance Fe storage, the pro-inflammatory action of Fe, and the low-grade inflammation of obesity which all play a role in the development of MetS and NAFLD [3,14,23,24,25,26,27].

Simple regression showed correlations between ferritin and z score and fibrinogen, blood lipids and transaminases; in addition overweight displayed a relationship with other components of MetS, BP and insulin resistance; sex-related differences were observed in the development of metabolic risk factors, such as previously reported [28].

These observations are consistent with previous reports which showed an association of Fe markers with inflammation, serum glucose levels and insulin sensitivity, blood pressure, MetS and also with cholesterol levels [25,29,30,31,32,33]. Similar observations were reported in obese teenagers, including increased transaminase levels; increased ferritin and transaminase levels are correlated [3,34,35,36,37,38,39,40]. Reduction of iron stores had positive influence on MetS and NAFLD; when occurring after a phlebotomy, these changes could be partly explained by its hemodynamic effects [41,42].

However, only a few reports attempted to dissociate the relative contributions of every risk factor to MetS and NAFLD [3,33,43].

Therefore, covariate regression analyses included sex and age as covariates; they displayed additive but independent effects between the three variables studied, ferritin, z score, and fibrinogen and risk factors; the main determinant appears to be obesity itself: z score was the only covariate to correlate with BP and insulin resistance; in addition to z score, ferritin displayed an independent relationship with blood lipids (triglycerides and cholesterol fractions), while fibrinogen had an inverse association with HDL concentrations. Concerning liver function, ferritin, z score and fibrinogen were independently predicative parameters of transaminase levels.

These multiple relationships reflect the complexity of pathogenicity of obesity complications.

Liver is an important site for iron, lipid, and glucose metabolism, and the main site for the interactions between their metabolic pathways. Increased iron storage has been linked with more advanced stages of NAFLD [44]. Increasing hepatic iron up-regulates the transcripts of several enzymes, including the rate limiting enzyme HMG CoA reductase, suggesting that hepatic iron loading increases liver cholesterol synthesis. The currently accepted hypothesis is that iron overload plays a role in the progression of NAFLD via increased inflammation and oxidative stress [14]; it is assumed that primary augmentation of oxidative stress is also a key mechanism underlying iron-induced insulin resistance [23]. Increased iron accumulation in liver in NAFLD might be due to the decrease in levels of the iron export protein ferroportin induced by the decrease of the iron regulatory peptide hepcidin [14]: liver synthesis of hepcidin is up-regulated in response to increased iron stores and inflammation; hepcidin enhances the degradation of ferroportin which allows Fe release [17,45,46]; increased expression of hepcidin and lower expression of ferroportin in patients with NAFLD could result in iron retention [46]. Iron overload could then generate more oxidative stress and inflammation [14]. It also may worsen insulin resistance by interfering with insulin receptor signaling and inhibiting the ability to burn carbohydrates in the liver and muscle.

Besides liver, adipocytes also have a function in insulin resistance: iron treatment increased lipolysis and impaired glucose uptake in response to insulin [47].

## 5. Conclusions

This study showed independent interactions between Fe status, inflammation and overweight in obese youth, and the occurrence of metabolic risk factors of MetS and NAFLD. Further studies using complementary parameters of Fe metabolism, such as sTfR and more hepcidin, owing to its central role in Fe regulation, should help to understand the mechanisms involved.

## References

[1] Pinhas-Hamiel O., Newfield R.S., Koren I., Agmon A., Lilos P., Phillip M. (2003). Greater prevalence of iron deficiency in overweight and obese children and adolescents. Int. J. Obes. Relat. Metab. Disord..

[2] Nead K.G., Halterman J.S., Kaczorowski J.M., Auinger P., Weitzman M. (2004). Overweight children and adolescents: A risk group for iron deficiency. Pediatrics.

[3] Mandato C., Lucariello S., Licenziati M.R., Franzese A., Spagnuolo M.I., Ficarella R., Pacilio M., Amitrano M., Capuano G., Meli R. (2005). Metabolic, hormonal, oxidative, and inflammatory factors in pediatric obesity-related liver disease. J. Pediatr..

[4] Moayeri H., Bidad K., Zadhoush S., Gholami N., Anari S. (2006). Increasing prevalence of iron deficiency in overweight and obese children and adolescents (Tehran Adolescent Obesity Study). Eur. J. Pediatr..

[5] Brotanek J.M., Gosz J., Weitzman M., Flores G. (2007). Iron deficiency in early childhood in the United States: Risk factors and racial/ethnic disparities. Pediatrics.

[6] Richardson M.W., Ang L., Visintainer P.F., Wittcopp C.A. (2009). The abnormal measures of iron homeostasis in pediatric obesity are associated with the inflammation of obesity. Int. J. Pediatr. Endocrinol..

[7] Amato A., Santoro N., Calabrò P., Grandone A., Swinkels D.W., Perrone L., Miraglia del Giudice E. (2010). Effect of body mass index reduction on serum hepcidin levels and iron status in obese children. Int. J. Obes..

[8] Cheng H.L., Bryant C., Cookn R., O’Connor H., Rooneyn K., Steinbeck K. (2012). The relationship between obesity and hypoferraemia in adults: A systematic review. Obes. Rev..

[9] Moschonis G., Chrousos G.P., Lionis C., Mougios V., Manios Y., Healthy Growth Study Group (2012). Association of total body and visceral fat mass with iron deficiency in preadolescents: The Healthy Growth Study. Br. J. Nutr..

[10] Baumgartner J., Smuts C.M., Aeberli I., Malan L., Tjalsma H., Zimmermann M.B. (2013). Overweight impairs efficacy of iron supplementation in iron-deficient South African children: A randomized controlled intervention. Int. J. Obes..

[11] Jehn M., Clark J.M., Guallar E. (2004). Serum ferritin and risk of the metabolic syndrome in U.S. adults. Diabetes Care.

[12] Choi K.M., Lee K.W., Kim H.Y., Seo J.A., Kim S.G., Kim N.H., Choi D.S., Baik S.H. (2005). Association among serum ferritin, alanine aminotransferase levels, and metabolic syndrome in Korean postmenopausal women. Metabolism.

[13] Neuschwander-Tetri B.A. (2007). Fatty liver and the metabolic syndrome. Curr. Opin. Gastroenterol..

[14] Ahmed U., Latham P.S., Oates P.S. (2012). Interactions between hepatic iron and lipid metabolism with possible relevance to steatohepatitis. World J. Gastroenterol..

[15] Anses Choix des Examens du Métabolisme du fer en cas de Suspicion de Carence en fer––Rapport D’Évaluation Technologique (in French).

[16] World Health Organization (2001). Iron Deficiency Anaemia—Assessment, Prevention and Control.

[17] Tussing-Humphreys L., Pusatcioglu C., Nemeth E., Braunschweig C. (2012). Rethinking iron regulation and assessment in iron deficiency, anemia of chronic disease, and obesity: Introducing hepcidin. J. Acad. Nutr. Diet..

[18] Brugnara C. (2013). Iron deficiency: What are the future trends in diagnostics and therapeutics?. Clin. Chem..

[19] Engle-Stone R., Nankap M., Ndjebayi A.O., Erhardt J.G., Brown K.H. (2013). Plasma ferritin and soluble transferrin receptor concentrations and body iron stores identify similar risk factors for irondeficiency but result in different estimates of the national prevalence of irondeficiency and iron-deficiency anemia among women and children in Cameroon. J. Nutr..

[20] Rolland-Cachera M.F., Cole T.J., Sempé M., Tichet J., Rossignol C., Charraud A. (1991). Body Mass Index variations: Centiles from birth to 87 years. Eur. J. Clin. Nutr..

[21] Hercberg S., Preziosi P., Galan P. (2001). Iron deficiency in Europe. Public Health Nutr..

[22] Cooper M., Greene-Finestone L., Lowell H., Levesque J., Robinson S. (2012). Iron sufficiency of Canadians. Health Rep..

[23] Whaley-Connell A., McCullough P.A., Sowers J.R. (2011). The role of oxidative stress in the metabolic syndrome. Rev. Cardiovasc. Med..

[24] Fumeron F., Pean F., Driss F., Balkau B., Tichet J., Marre M., Grandchamp B. (2006). Ferritin and transferrin are both predictive of the onset of hyperglycemia in men and women over 3 years: The data from an epidemiological study on the Insulin Resistance Syndrome (DESIR) study. Diabetes Care.

[25] Wrede C.E., Buettner R., Bollheimer L.C., Scholmerich J., Palitzsch K.D., Hellerbrand C. (2006). Association between serum ferritin and the insulin resistance syndrome in a representative population. Eur. J. Endocrinol..

[26] Dongiovanni P., Fracanzani A.L., Fargion S., Valenti L. (2011). Iron in fatty liver and in the metabolic syndrome: A promising therapeutic target. J. Hepatol..

[27] Valenti L., Swinkels D.W., Burdick L., Dongiovannin P., Tjalsman H., Mottan B.M., Bertelli C., Fatta E., Bignamini D., Rametta R. (2011). Serum ferritin levels are associated with vascular damage in patients with nonalcoholic fatty liver disease. Nutr. Metab. Cardiovasc. Dis..

[28] Harrison-Findik D.D. (2010). Gender-related variations in iron metabolism and liver diseases. World J. Hepatol..

[29] Fernandez-Real J.M., Lopez-Bermejo A., Ricart W. (2005). Iron stores, blood donation, and insulin sensitivity and secretion. Clin. Chem..

[30] Gonzalez A.S., Guerrero D.B., Soto M.B., Diaz S.P., Martinez-Olmos M., Vidal O. (2006). Metabolic syndrome, insulin resistance and the inflammation markers C-reactive protein and ferritin. Eur. J. Clin. Nutr..

[31] Zelber-Sagi S., Nitzan-Kaluski D., Halpern Z. (2007). NAFLD and hyperinsulinemia are major determinants of serum ferritin levels. J. Hepatol..

[32] Lecube A., Hernández C., Pelegrí D., Simó R. (2008). Factors accounting for high ferritin levels in obesity. Int. J. Obes. (Lond.).

[33] Sun L., Franco O.H., Hu F.B., Cai L., Yu Z., Li H., Ye X., Qi Q., Wang J., Pan A. (2008). Ferritin concentrations, metabolic syndrome, and type 2 diabetes in middle-aged and elderly chinese. J. Clin. Endocrinol. Metab..

[34] Dubern B., Girardet J.P., Tounian P. (2006). Insulin resistance and ferritin as major determinants of abnormal serum aminotransferase in severely obese children. Int. J. Pediatr. Obes..

[35] Ford E.S., Ajani U.A., Mokdad A.H. (2005). The metabolic syndrome and concentrations of C-reactive protein among U.S. youth. Diabetes Care.

[36] Skinner A.C., Steiner M.J., Henderson F.W., Perrin E.M. (2010). Multiple markers of inflammation and weight status: Cross-sectional analyses throughout childhood. Pediatrics.

[37] Syrovatka P., Kraml P., Potockova J., Fialova L., Vejrazka M., Crkovska J., Andel M. (2009). Relationship between increased body iron stores, oxidative stress and insulin resistance in healthy men. Ann. Nutr. Metab..

[38] Tussing-Humphreys L.M., Liang H., Nemeth E., Freels S., Braunschweig C.A. (2009). Excess adiposity, inflammation, and iron-deficiency in female adolescents. J. Am. Diet. Assoc..

[39] Cepeda-Lopez A.C., Osendarp S.J., Melse-Boonstra A., Aeberli I., Gonzalez-Salazar F., Feskens E., Villalpando S., Zimmermann M.B. (2011). Sharply higher rates of iron deficiency in obese Mexican women and children are predicted by obesity-related inflammation rather than by differences in dietary iron intake. Am. J. Clin. Nutr..

[40] Hiura M., Kikuchi T., Nagasaki K., Uchiyama M. (2003). Elevation of serum C-reactive protein levels is associated with obesity in boys. Hypertens. Res..

[41] Houschyar K.S., Lüdtke R., Dobos G.J., Kalusn U., Broecker-Preussn M., Rampp T., Brinkhaus B., Michalsen A. (2012). Effects of phlebotomy-induced reduction of body iron stores on metabolic syndrome: Results from a randomized clinical trial. BMC Med..

[42] Sumida Y., Kanemasa K., Fukumoto K., Yoshida N., Sakai K., Nakashima T., Okanoue T. (2006). Effect of iron reduction by phlebotomy in Japanese patients with nonalcoholic steatohepatitis: A pilot study. Hepatol. Res..

[43] Escobar-Morreale H.F., Villuendas G., Botella-Carretero J.I., Sancho J., San Millan J.L. (2003). Obesity, and not insulin resistance, is the major determinant of serum inflammatory cardiovascular risk markers in pre-menopausal women. Diabetologia.

[44] Kowdley K.V., Belt P., Wilson L.A., Yeh M.M., Neuschwander-Tetri B.A., Chalasani N. (2012). Serum ferritin is an independent predictor of histologic severity and advanced fibrosis in patients with nonalcoholic fatty liver disease. Hepatology.

[45] Aigner E., Theurl I., Theurl M., Lederer D., Haufe H., Dietze O., Strasser M., Datz C., Weiss G. (2008). Pathways underlying iron accumulation in human nonalcoholic fatty liver disease. Am. J. Clin. Nutr..

[46] Gabrielsen J., Gao Y., Simcox J.A., Huang J., Thorup D., Jones D., Cooksey R.C., Gabrielsen D., Adams T.D., Hunt S.C. (2012). Adipocyte iron regulates adiponectin and insulin sensitivity. J. Clin. Investig..

[47] Datz C., Felder T.K., Niederseer D., Aigner E. (2013). Iron homeostasis in the Metabolic Syndrome. Eur. J. Clin. Investig..

